# Proteomic analysis of rat cartilage: the identification of differentially expressed proteins in the early stages of osteoarthritis

**DOI:** 10.1186/s12953-014-0055-0

**Published:** 2014-11-18

**Authors:** Nancy Marbella Parra-Torres, Febe Elena Cázares-Raga, Juan Bautista Kouri

**Affiliations:** Departamento de Infectómica y Patogénesis Molecular, Centro de Investigación y de Estudios Avanzados, Instituto Politécnico Nacional (CINVESTAV-IPN), México, DF México

**Keywords:** Osteoarthritis, Cartilage, Proteomics

## Abstract

**Background:**

Osteoarthritis (OA) is a chronic degenerative disease of the articular cartilage, and its diagnosis is based on symptoms and radiological signs that are only present in the late stages of the disease. Due to the limitations in diagnosing OA before the onset of symptoms, such as pain, little is known about the molecular mechanisms involved in the pathogenesis of OA. Experimental OA models are often used to study the kinetics of the progression of this disease. In this report, we conducted a proteomic study of osteoarthritic cartilage during the early stages of OA using an experimental rat model.

**Results:**

Ten proteins that are differentially expressed under early OA conditions were identified by 2-DE and MALDI-TOF/MS. These proteins mediated many processes, such as glycolysis and energy production (Nme2 and Pnp), cartilage matrix (Col2a1), transcription and protein synthesis (Eef1a1 and DJ-1), signal transduction (CaM and Pebp1), transport (Alb and Hba1), and latexin (Lxn). In addition, changes in Lxn expression in early OA were observed and validated by western blot and immunofluorescence analysis.

**Conclusions:**

The proteins that we identified indicate that energy metabolism, cartilage matrix remodelling, and protective cellular mechanisms are associated with early OA. In addition, latexin expression during the early stages of OA could be implicated in cartilage repair.

**Electronic supplementary material:**

The online version of this article (doi:10.1186/s12953-014-0055-0) contains supplementary material, which is available to authorized users.

## Background

Osteoarthritis (OA) is a chronic degenerative joint disease that is characterized by extracellular matrix (ECM) degradation and cell death, resulting in the gradual loss of articular cartilage integrity [1-7]. In particular, certain ECM components, such as collagen (types II, VI, and X), proteoglycans (aggrecan, decorin, and lumican), and non-collagenous proteins (annexin and fibronectin) are degraded during OA pathogenesis, generating fragments that negatively regulate metabolic processes in chondrocytes [8]. The failure of chondrocytes to maintain a proper balance between the synthesis and degradation of the ECM has been suggested to result in cartilage degeneration during OA pathogenesis [5,9-11].

However, little is known about the molecular mechanisms involved in the early stages of OA due to the limitations in detecting the early symptoms of the disease before the onset of pain, which only manifests during the late stages. The use of proteomic technologies then allows the identification of novel biomarkers in tissues and cells as indicators of normal and pathological processes, such as OA [12-16]. Furthermore, these approaches are being applied to study the molecular basis of OA etiology and the mechanisms that mediate cartilage regeneration and joint destruction in the later stages of OA [16].

Previous works used two-dimensional electrophoresis (2-DE) to identify certain proteins, such as the precursor of collagens I and VI, annexin A1 (ANNX 1), and phosphatidylethanolamine-binding protein 1 (Pebp1), which were differentially expressed in chondrocytes from healthy and osteoarthritic human cartilage [16]. However, these changes were observed only for proteins in the advanced stages of OA, which precludes the early diagnosis of disease [16,17]. Thus, experimental OA models are often used to study the kinetics of the processes that mediate the development of OA [7].

Initially, our group generated a proteomic map of normal rat articular cartilage, in which the expression of latexin (Lxn), a carboxypeptidase A inhibitor, was identified for the first time [18]. In this work, we performed a proteomic study of osteoarthritic cartilage during the early stages of an experimental OA rat model [7]. Ten proteins were differentially expressed over time and identified by MALDI-TOF/MS. Notably, Lxn was one of the proteins that was modified early as detected in the OA cartilage of rats.

## Results and discussion

Our previous reports examined the histological and molecular changes of cartilage during OA pathogenesis in an experimental animal model of OA, and showed that our model can be evaluated according to Mankin’s histological grade parameters [2,5,7,19]. Twenty days after induction, our rat OA animal model reproduces the changes that occur in human cartilage in the late stages of OA [20]; which including fibrillation the loss of tissue cartilage mineralization and the formation of clusters [5,7,21-25]. However, using our animal model also allows OA kinetic changes to be followed starting in the early stages of OA; including the initial fibrillation of the cartilage surface area and superficial zone surrounding chondrocytes and the initial formation of cell clusters. These changes were observed after 5 days of OA induction [7,19,25]. Therefore and because these changes are characteristic of early OA, we studied the changes in the protein profile at 3, 5, and 10 days after the induction of OA.

### Identification of early macroscopic changes in articular cartilage of normal, sham, and OA-induced rats

**Figure 1:**
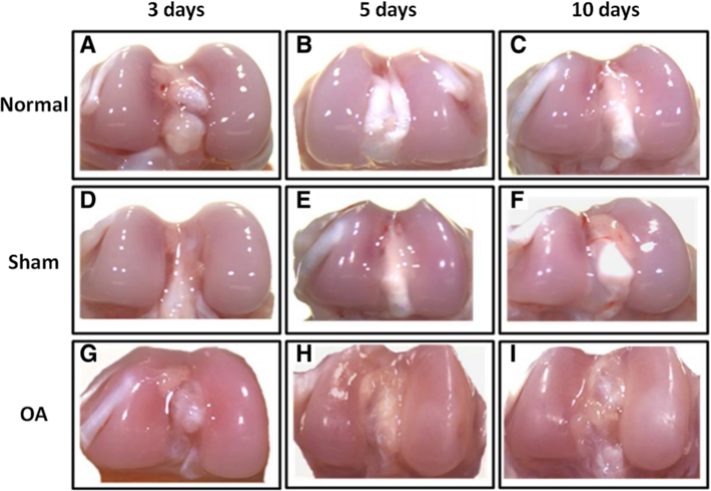
**Macroscopic changes in the articular cartilage of rat during the early stages of OA.** Femoral condyles from normal, sham, and early induced OA as viewed with a stereomicroscope. **(A-C)** Femoral condyles from normal, **(D-F)** sham, and **(G-I)** OA-induced (OA 3, 5, and 10 days) rats.

The sham surgical procedure did not significantly change the cartilage (Figure 1A-C): the tissues were smooth with a shiny pink surface (Figure 1D-F). In contrast, the cartilage from OA-induced animals underwent evident changes starting 3 days after OA induction, showing an opaque appearance, rough surface, and a white colour in the affected area, and these changes were more pronounced during OA pathogenesis (right condyle) (Figure 1G-I). These results demonstrate that a partial menisectomy and high-impact exercise cause damage the articular cartilage morphology, which causes joint deformity and induces OA.

### Differential protein expression between normal and OA cartilage during early stages in a rat model

**Figure 2:**
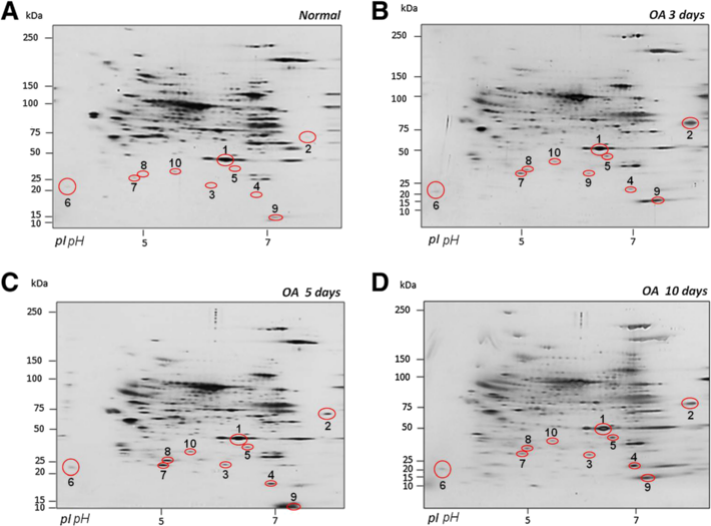
**Representative 2-DE proteomic profiles of normal and OA cartilage during the early stages (3, 5, and 10 days).** The proteins were resolved on IPG strips at pH 3–10 NL using an SDS-PAGE gradient (5-20%). The gels were silver-stained. **(A)** Profiles from normal conditions. **(B)** OA 3 days. **(C)** OA 5 days. **(D)** OA 10 days. The selected spots are numbered and marked with red circles.

**Figure 3:**
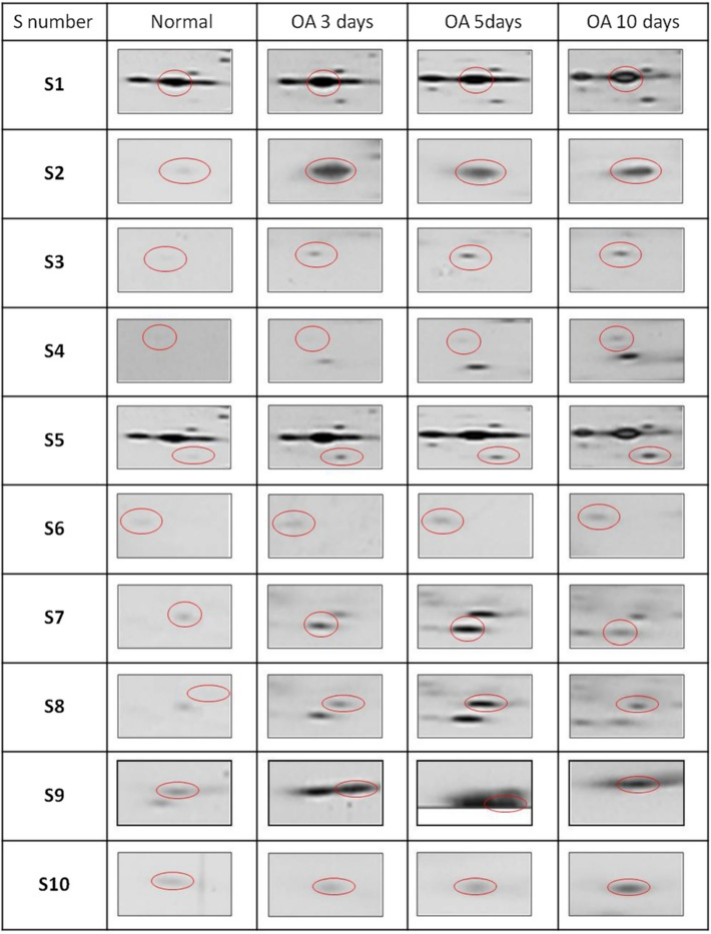
**Differentially expressed proteins during early OA by 2-DE and MALDI-TOF/MS.** Ten proteins were differentially expressed during the early stages of OA (3, 5, and 10 days), compared with normal levels (see Table 1).

The cartilage proteins were purified from the joints of rats during the early experimental OA stage (normal, and OA at 3, 5, and 10 days) and resolved by 2-DE; 280 ± 17 spots were detected in the gels for each condition, and four spots with equivalent expression in all samples were selected as internal controls. The spots were consistent between duplicates for each condition, indicating reproducibility. In OA-induced cartilage, we observed 10 spots in the early OA stages with differential expression versus the normal levels (Figure 2). Moreover, we observed patent differences in the spot volumes between 3, 5, and 10 days after OA induction (Figure 3).

The expressions of spot 1 (S1) and S2 increased and peaked after at 3 days of OA induction compared with the normal samples. In addition, S3, S7, and S8 were significantly up-regulated after 5 days, whereas S4, S5, S9, and S10 peaked at 10 days (Figure 3). The differences in the spot intensity were quantified using the relative volume percentage (vol%) and compared with the control values. The images of the silver-stained gels were analysed using ImageMaster 2D Platinum, version 7.0 (Tukey-Kramer test, Table 1).

**Table 1:** **Functional classification of differentially expressed proteins in normal articular cartilage and during early OA in rat identified by 2-DE and MALDI-TOF/MS**

**Spot** **no.** ^**a)**^	**Protein** **name** ^**b)**^	**Description**	**Accession** **no.** ^**b)**^	**Theor M**_**r/**_ **/p** ***I*** ^**c)**^	**Exp. M**_**r**_ **/p** ***I*** ^**d)**^	**% Seq** **Cov.** ^**e)**^	**Match**	**Score** ^**f)**^	**Proteins expression compared with Normal samples (% increase)** ^**g)**^		
		**Protein**					**Pept.**				
									**OA 3 days**	**OA 5 days**	**OA 10 days**
		**Extracellular Matrix**									
**S1**	Col2α1	Collagen alpha-1 (II) chain precursor	P05539	38/8.46	30-32/6.6-6.7	12	16	84	**22**	**15**	**12**
		**Transcription, protein synthesis**									
**S2**	Eef1α1	Elongation factor 1-alpha 1	P62630	50.11/9.1	50.11/9.1	32	13	37	**499**	**407**	**314**
**S3**	DJ-1	Protein DJ-1	88767	19.9/6.32	19.9/6.3	54	9	85	**138**	**313**	**271**
		**Glycolysis and energy production**									
**S4**	Nme2	Nucleoside diphosphate kinase	P19804	17.28/6.9	17.3/6.9	87	16	187	**127**	**515**	**700**
**S5**	Pnp	Purine nucleoside phosphorylase	P85973	32.3/6.46	32.32/6.4-6.5	43	8	49	**243**	**277**	**384**
		**Signal transduction**									
**S6**	CaM	Calmodulin	P62161	16.7/4.09	16.8/5.47	63	8	101	**124**	**130**	**185**
**S7**	Pebp1	Phosphatidylethanolamine binding protein 1	P31044	20.6/5.48	17.18/4.9-5.5	52	7	76	**54**	**92**	**29**
		**Transport**									
**S8**	Alb	Serum albumin (n-terminal fragment)	P02770	65.9/5.8	68.6/6.09	24	12	105	**435**	**853**	**568**
**S9**	Hba1	Haemoglobin subunit alpha-1/2	P01946	15.1/7.93	19.7/6.32	69	6	88	**231**	**157**	**284**
		**Others**									
**S10**	Lxn	Latexin	Q64361	25.5/5.77	25/5.8	48	11	118	**20**	**29**	**54**

^a)^Spot number as indicated in Figure 2.

^b)^Protein name and accession number according UniprotKB/Swiss-prot database of *Rattus norvegicus* species.

^c)^Theoretical M_r_ and p*I* according to protein sequence.

^d)^Experimental M_r_ and p*I* calculated by analysis of the gel image with ImageMaster 2D Platinum 7.0 software.

^e)^Sequence coverage for identified protein (peptide confidence ≥95) in relation on the full sequence.

^f)^The Score value listed within the Query table is the Ion Score. Ion score is −10*Log (P), where P is the probability that the observed match is random event.

^g)^% Relative volume by condition in early OA vs Normal condition (Tukey-Kramer test, *p* ≤0.001). The percentage of increase of the protein expression is represented by bold numbers.

### Identification and functional classification of differentially expressed proteins

The 10 differentially expressed proteins were excised, digested, and identified by MALDI-TOF/MS. Table 1 shows the changes in the expression of these proteins on a 2-DE reference map (Additional file 1: Figure S1). Based on their known functions, these proteins were classified into 7 groups: glycolysis and energy production (Nme2 and Pnp), cartilage matrix (Col2a1), transcription and protein synthesis (DJ-1 and Eef1a1), signal transduction (CaM and Pebp1), transport (Alb and Hba1), and latexin.

Two proteins mediated metabolism and energy production (Pnp and Nme2) [26,27]. Pnp has been implicated in purine metabolism, and its expression is linked to the accumulation of toxic levels of deoxyguanosine triphosphate (dGTP) which induces apoptosis in T lymphocytes [28]. Nme2 catalyses the transfer of terminal phosphate groups of nucleoside 5′-triphosphate to nucleoside 5′-diphosphate, which mediates the biosynthesis of ribo- and deoxyribonucleoside triphosphates, except ATP [27]; it also regulates a wide range of cellular functions, including proliferation, differentiation, and neoplastic transformation [29]. The increase in both proteins during early OA could implicate them in metabolic and purine salvage pathways as a consequence of the increase in catabolism during OA pathogenesis.

Type II collagen is the principal component of articular cartilage and comprises its basic architecture, and it is the chief constituent of collagens [30,31]. However, in the late stages of OA, type II collagen is substituted by other collagens, such as types I and X, due to the catabolic activity of chondrocytes [16,32]. Our results showed that the type II collagen levels increased 3 and 5 days after OA induction, which might be related to a reparative cartilage phase. This positive regulation during OA might be a self-protective response of chondrocytes to changes in the pericellular environment that wane during at later stages [11,16,28,31,32].

Other proteins that regulate the synthesis of proteins involved in ECM remodelling via MMP-1, MMP-3, and MMP-13 activation [33], such as Pebp1 and CaM, are also up regulated during the early stages of OA. Pebp1 is a Raf kinase inhibitor that has been identified in the proteome of normal human articular cartilage, whereas it has shown decreased expression in the late stages of OA [8,16,18]. Our results suggested that Pebp1 participates in ECM remodelling during early OA, possibly via the regulation of downstream signalling pathways.

CaM regulates signal transduction, forming calcium-calmodulin complex- dependent protein kinase II (CaMKII) [34]. In addition, the CaMKII complex has been implicated as a signalling receptor for acid-N-methyl-D-aspartate (NMDA) and is activated as part of the mechanotransduction response of normal human articular chondrocytes to mechanical stimulation. However, this route is not activated after stimulation in OA chondrocytes [35]. Thus, the dysregulation of CaMKII signaling has been suggested to be significant for the onset and progression of OA.

In addition, DJ-1 and Eef1α1 were identified as proteins that mediate the transcription and synthesis of others proteins. DJ-1 has several functions, such as protection against oxidative stress and cell death [36]. Furthermore, it regulates transcription as a co-activator [37,38]. The chondrocyte apoptosis induced by reactive oxygen species (ROS) has been considered important in OA pathogenesis [39,40]. Our results have demonstrated that DJ-1 is up-regulated in the early stages of OA, which suggested its involvement in the regeneration of cartilage due to the prevention chondrocyte death via the removal of ROS. Eef1α1 is involved in translation [41] and also increases in expression, indicating that chondrocytes up-regulate translation machinery during early OA to synthesize proteins and repair damaged cartilage [5,10].

Hba1 and Alb, which mediate oxygen transport and osmotic pressure, have been previously detected in cartilage. Both proteins were up-regulated but their functions in OA cartilage remain unknown [8,18].

Latexin (Lxn), a carboxypeptidase A inhibitor, was up-regulated during early OA. The expression of this protein has previously been reported in rat cartilage [18], thus, we focused on latexin analysis.

### Differential expression of Lxn in early OA-induced cartilage in rats

**Figure 4:**
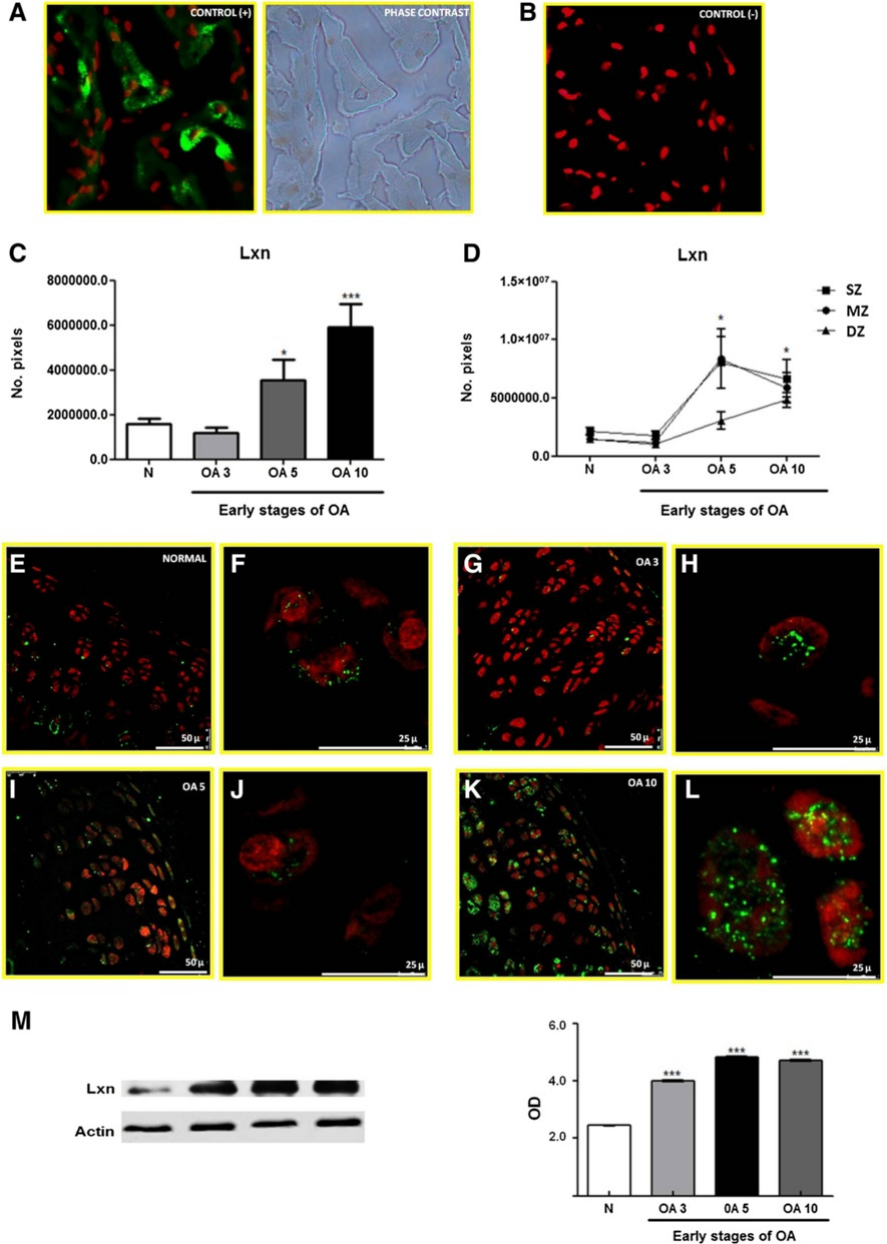
**Expression and localization of Lxn in normal articular cartilage during the early stages of OA in a rat model. (A)** Positive control and phase contrast microscopy for Lxn expression in rat heart tissue. **(B)** Negative control for Lxn expression in rat heart tissue. **(C)** Number of pixels, expressed as area of Lxn in the total cartilage. **(D)** Expression of Lxn in the 3 zones (SZ, MZ, and DZ) in normal and osteoarthritic cartilage. **(E, G, I, and K)** Lxn expression in normal articular cartilage and OA at 3, 5, and 10 days **(F, H, J, and L)**, perinuclear and cytoplasmic localization of Lxn in normal articular cartilage and OA at 3, 5, and 10 days (amplification of panel **E**, **G**, **I**, and **K**). **(M)** Representative western blot of Lxn expression in normal and OA cartilage during early stages (3, 5, and 10 days). The densitometry graph shows the protein levels of Lxn measured by optical density (OD) normalized to β-actin. The results show the means ± S.E.M. of 3 independent experiments using a Tukey-Kramer multiple comparison test. Significant differences are represented by asterisks (*p < 0.05, **p < 0.01, ***p < 0.001).

A previous study reported Lxn expression in the articular cartilage [18] of rats. Lxn mediates the development of skeletogenesis and the growth plate [42]. The increase in the rise Lxn levels during early OA found by the proteomic analysis suggests that it mediates OA pathogenesis. Thus, we examined Lxn with a proteomic analysis to validate these results during early OA. We utilized immunofluorescence and WB to analyse its expression and localization in articular cartilage. Because we have previously observed that the normal and sham controls are similar [19,22] we only used the normal control for the validation experiments. Figure 4 shows the positive and negative controls for Lxn in rat heart tissue (A and B). In addition, Figure 4A includes phase contrast microscopy to show the Lxn localization in the tissue.

In normal rat articular cartilage, Lxn is primarily expressed in the chondrocytes of 3 cartilage zones—the surface zone (SZ), middle zone (MZ), and deep zone (DZ)—at all time points (Figure 4E, G, I, and K). The Lxn levels were higher in osteoarthritic cartilage 5 and 10 days after OA was induced (Figure 4C and D). The distribution of Lxn, was granular in the cytoplasm of most chondrocytes and arranged in the perinuclear MZ and DZ in some chondrocytes (Figure 4F, H, J, and L).

The expression of Lxn was also analysed by WB under normal conditions and during early OA. As shown in Figure 4M, the expression of Lxn was up regulated after 3 days and maximized 10 days after OA induction compared to normal cartilage. These data corroborated our 2-DE findings, which validated the use of these tools to examine the differential expression of proteins during early OA.

The expression of Lxn was also analysed by WB under normal conditions and during early OA. As shown in Figure 4M, Lxn was up-regulated 149% from the normal cartilage level after 3 days, and this increase reached 215% and 207% after 5 and 10 days, respectively. These data corroborated our 2-DE findings, which validated the use of these tools to examine the differential expression of proteins during early OA; when Lxn was up-regulated 20% after 3 days, 29% after 5 days, and 54% after 10 days of OA induction. The increases observed by WB were more pronounced that those observed by 2-DE, likely due to antibody specificity.

The expression of Latexin (Lxn), an inhibitor of carboxipeptidase A, was increased during the early stages of OA, which suggested that it acted as a regulator of carboxypeptidase activity. This effect may have reduced the ability of these enzymes to bind to substrates, which inhibited degradation. This protective mechanism of the chondrocytes might prevent mechanical damage to cartilage which is involved in the development of OA. Lxn might also participate in other cartilage repair processes, such as the proliferation of chondrocytes and cartilage regeneration, in response to the damage that is incurred during early OA [42,43]. Recently, Lxn was reported to be localized in the nuclei and cytoplasm of proliferating and prehypertrophic chondrocytes during skeletogenesis and skeletal regeneration [42]. Latexin was also identified as negative regulator of stem cell replication and increased apoptosis [44]. Previous works have shown that chondrocytes in the cartilage of older people remain in a resting phase of the cell cycle, which limits their potential for growth. This arrest has also been associated with changes in telomere length [45]. Thus, Lxn may govern senescence mechanisms in chondrocytes [1].

## Conclusion

The rat model of induced osteoarthritis has allowed us to demonstrate the differential expression of proteins during the early stages of OA with a proteomic analysis. The changes in the expression of some proteins during the early stages of OA suggested its participation in the reparative phase of the cartilage because most of the identified proteins were involved in various reparative cell processes such as the remodelling of the ECM, energy metabolism, and protective cellular mechanisms.

In addition, this study examined the expression and immunolocalization of Lxn, which appeared to play a role in the cartilage repair phase of early OA as an inhibitor of degradative enzymes. Although additional work is required to understand the relationship between these proteins and early cartilage injury, our results have enabled us to identified novel proteins involved in the start of OA pathogenesis, which could guide the discovery of biomarkers for this disease in humans.

## Materials and methods

### Tissue sampling

Cartilage samples were obtained from the knees of 159 normal male Wistar rats (130–150 g) and 1017 rats with OA that were induced by partial menisectomy of the right hind leg, followed by high-impact exercises 3, 5, and 10 days after OA induction (early OA). This procedure has been described elsewhere [2,5,7,19] and shown to reproduce the cartilage changes observed during the late stages of human OA 20 days after OA induction in rats [20]. These changes include fibrillation, a loss of tissue, cartilage mineralization and the formation of clusters [5,7]. Cartilage samples were also obtained from 240 sham rats (incision without menisectomy or exercises). All procedures involving animals were approved by our institutional committee (CICUAL-Cinvestav-IPN) and performed following the animal facility regulations and Mexican official regulatory Guideline for the Care and Use of Laboratory Animals; NOM-069-ZOO-1999.

### Protein extraction and preparation

A pool of cartilage samples from the knees of 30 normal rats, 120 sham rats, and 120 rats with conditionally induced OA (3, 5, and 10 days after OA induction) were used by independent experiment. The proteins were extracted based on selective extraction as reported by Vincourt [13] with some modification [18]. Briefly, samples were frozen in liquid nitrogen, mechanically pulverized, suspended in extraction buffer [500 mM NaCl, 50 mM HEPES pH 7.2, complete protease inhibitor cocktail, (Roche Applied Science) and homogenized. In addition, the samples were sonicated (POLYTRON® PT 2100) (10 strokes at 15,000 × g) and then stored overnight at 4°C. The insoluble material was then removed by centrifugation (6,000 × g, at 4°C for 7 min), the supernatant was recovered, and cetylpyridinium chloride (CPC) 1% (w/v) was added to remove proteoglycans (PGs) followed by another centrifugation (6,000 × g, at 4°C for 7 min) [46]. To remove the lipids and salts, the supernatant was precipitated with methanol (400 μL methanol/100 μL sample) and centrifuged (14,000 × g, for 30 min), and the liquid phase was removed. This procedure was repeated twice. The pellet was resuspended in 2-DE sample buffer [7 M urea, 2 M thiourea, 4% (w/v) CHAPS, 2% (v/v) immobilized pH gradient (IPG) buffer, pH 3–10 (GE Healthcare Life Sciences, Sweden) and 40 mM dithiothreitol (DTT)] and supplemented with protease inhibitors [13,14]. In addition, the 2D Clean-Up Kit (GE Healthcare) was used for selective protein precipitation and cleaning. The precipitate was resuspended in rehydration solution [7 M urea, 2 M thiourea, 2% (w/v) CHAPS, 0.5% (v/v) IPG buffer, pH 3–10, and 154 mM DTT, and 0.001% bromophenol blue] supplemented with protease inhibitors. The protein concentration was measured with a Bradford assay [19]. All experiments were performed in independent duplicates.

### Two-dimensional electrophoresis (2-DE)

Protein extract (300 μg) resuspended in rehydration solution (250 μL) was used to rehydrate Immobiline DryStrip gels (IPG strips) that were 13 cm in size (GE, Healthcare) at pH 3–10 NL for 12 h at room temperature. Electrofocusing was performed in an Ettan IPGphor 3 (GE Healthcare, USA) per the manufacturer’s protocols. The disulphide bonds in proteins were reduced and alkylated using DTT and iodacetamide, respectively. The proteins were silver-stained immediately after to the second-dimension run on an SDS-PAGE gradient (5–20%) [18]. These experiments were performed in duplicate, and technical replicas (data not shown) were only implemented for controls.

### Image acquisition and data analysis

Digital images of 2-DE gels were obtained using an ImageQuant LAS 4000 System (GE Healthcare) and analysed using ImageMaster 2D Platinum, version 7.0 (GE Healthcare Life Sciences, Switzerland) to measure the protein expression levels. The spots were counted and compared automatically with subsequent manual correction. For the semiquantitative comparison, the optical densities were automatically detected; the spots were selected, the confidence interval for the difference between two means was obtained (data not shown) and analysed with a Tukey-Kramer multiple comparison test with at *p* ±0.001.

### Spot excision and trypsin digestion

The spots were processed after excision. The samples were dehydrated by incubation in 100 μL acetonitrile for 5 min at room temperature. The supernatant was then removed, and the gel fragments were allowed to dry. The peptides were digested as previously reported [47]. Briefly, dry polyacrylamide pieces were prepared for in-gel digestion with 0.020 μg modified trypsin (sequencing-grade, Roche Molecular Biochemicals) overnight at 37°C. The resulting peptides were extracted twice with ACN/TFA solution [50% w/v acetonitrile (ACN)/% w/v trifluoroacetic acid (TFA)], incubated for 10 min at room temperature and centrifuged at 14,000 × g for 30 sec. The peptide extracts were combined and reduced/desalted using C18-ZipTips (Millipore Corporation, Bedford, MA, USA).

### Mass spectrometry by MALDI

The resulting peptide extracts were analysed by MALDI-TOF/MS with a Voyager DE Pro mass spectrometer in the linear mode (Applied Biosystems) at the Protein Core Facility, Columbia University Medical Center, New York, USA. To identify the proteins by PMF, the molecular mass of each tryptic fragment was searched against the National Center for Biotechnology *Rattus norvegicus* database using the MASCOT program (http://www.matrixscience.com). A confidence interval ≥99% was used to identify the proteins (Unused ProtScore > 2.0). The possible oxidation of methionine residues, and carbamidomethylation at cysteine residues as variable modifications were considered. A maximum of one missed tryptic cleavage *per* protein was allowed in the database search. A mass accuracy of 100 ppm was used for MS. For positive identifications, a MASCOT score > 56 was considered significant (*p* < 0.05).

### Western blot

The cartilage samples of normal and OA-induced rats (3, 5, and 10 days) were frozen in liquid nitrogen, mechanically pulverized, suspended in lysis buffer (25 mM Tris–HCl, pH 7.6, 150 mM NaCl, 1% NP-40, 1% sodium deoxycholate, 0.1 % SDS) with complete protease inhibitor cocktail, and homogenized in a polytron tissue grinder (POLYTRON® PT 2100). The samples were shaken for 2 h at 4°C and clarified by centrifugation at 10,000 × g at 4°C for 5 min. The protein concentration was measured with a Bradford assay. Subsequently, the obtained samples were dissolved in Laemmli buffer (which contained 1% SDS and β-mercaptoethanol) and boiled for 5 min. SDS-PAGE was performed using 5–20% gradient gels of 7 cm and 80 μg of protein per lane, after which the proteins were transferred onto nitrocellulose membranes by wet transfer at 350 mA for 2.5 h.

The membranes were blocked with 5% non-fat dry milk in Tris-buffered saline (TBS), pH 7.5, containing 0.1% Tween 20 (TBS-T) for 2 h at 37°C with gentle shaking and incubated overnight at 4°C with rabbit polyclonal anti-Lxn (1:1000; sc-47089, Santa Cruz Biotechnology, Santa Cruz, CA, USA). Peroxidase-conjugated goat anti-rabbit was used as the secondary antibody (1:40,000; Jackson Immunoresearch Laboratories Inc. West Grove, PA, USA) for 2 h at room temperature. These experiments were performed in triplicate.

### Immunonofluorescence (IF)

To analyse the macroscopic characteristics of articular cartilage, femoral condyles were obtained from normal and OA-induced rats (3, 5, and 10 days) and observed under a stereomicroscope (E2 4D Leica, Heidelberg, Germany). The cartilage samples were then cryopreserved with 10% PBS-sucrose for 24 h, cryosectioned (Leica CM 1100; Heerbrugg, Switzerland), mounted on gelatine-coated slides, and stored at −20°C for 2 days. The samples were hydrated in PBS, treated with 0.2% Tween 20 in PBS for 10 min, and preincubated with 0.2% IgG-free bovine serum albumin (Sigma Chemical, Germany) for 20 min at room temperature. The sections were incubated overnight at 4°C with anti-Lxn goat polyclonal antibody (1:70, sc-47089, Santa Cruz Biotechnology, Santa Cruz, CA, USA) followed by incubation with fluorescein isothiocyanate (FITC)-tagged anti-goat IgG (1:50 Zymed Laboratories, South San Francisco, CA) for 1 h at room temperature. The nuclei were counterstained with propidium iodide for 1 min (1:3000; Vector Laboratories, Burlingame, CA), and the samples were mounted with Vectashield. As a negative control, the primary antibody was omitted. Rat heart tissue was used as a positive control. The sections were analysed with confocal microscopy (TCP-SP2, Leica, Heidelberg, Germany). These experiments were performed in triplicate.

### Statistical analysis

A statistical analysis was performed using pools of 30 rats by condition (normal, and 3, 5 and 10 days of OA induction) for western blot in three independent assays. The immunoreactions were visualized by chemiluminescence (ECL Plus western blotting detection system, GE Healthcare, Buckinghamshire, UK); the band intensities obtained from the western blot assays were quantified by optical densitometry. The relative intensity was expressed in arbitrary optical density units (OD). In all cases the statistical analysis was performed using a Tukey-Kramer multiple comparison test. Three rats were used for each condition for IF. Three sections were obtained from each rat. In each rat, 3 sections and 3 fields were randomly selected and analised; this procedure was performed in triplicate. The Leica LAS AF Lite confocal program was used to quantify the immunolabelling, wherein the fluorescence intensities were measured in each zone based on the number of pixels per area.

## Additional file

Additional file 1: Figure S1. Representative 2-DE proteomic maps of identified proteins in OA cartilage during early stages (3, 5, and 10 days). **(A-B)** Samples of OA cartilage at 3 days (duplicate experiment). Proteins were resolved on IPG strips pH 3–10 NL (5-20% SDS-PAGE gradient gels, gels were silver-stained). The spots were marked with arrowheads according to the database [UniProt Knowledge base (UniProtKB)/Swiss-Prot] for Rattus norvegicus species.

## Abbreviations

OA: Osteoarthritis
ECM: Extracellular matrix
2-DE: Two-dimensional electrophoresis
ANNX 1: Annexin 1
Pebp1: Phosphatidylethanolamine binding protein 1
Lxn: Latexin
CPC: Cetylpyridinium chloride
PGs: Proteoglycans
DTT: Dithiothreitol
ACN: Acetonitrile
TFA: Trifluoroacetic acid
MS: Mass spectrometry
FITC: Fluorescein isothiocyanate
DJ-1: Protein DJ-1
Alb: Serum albumin (n-terminal fragment)
Nme2: Nucleoside diphosphate kinase
Pnp: Purine nucleoside phosphorylase
CaM: Calmodulin
Hba1: Hemoglobin subunit alpha-1/2
Col2α1: Collagen alpha-1 (II) chain precursor
Eef1α1: Elongation factor 1-alpha 1
Vol%: % Relative volume
dGTP: Deoxyguanosine triphosphate
MMP1: Matrix metalloprotease 1
MMP-3: Matrix metalloprotease 3
MMP-13: Matrix metaloprotease 13
CaMKII: Calcium-calmodulin complex-dependent
NMDA: Acid-N-methyl-D-aspartate
ROS: Reactive oxygen species
ZS: Surface zone
ZM: Middle zone
ZP: Deep zone

## References

[1] Liu Q, Yu L, Gao J, Fu Q, Zhang J, Zhang P, Chen J, Zhao S (2000). Cloning, tissue expression pattern and genomic organization of latexin, a human homologue of rat carboxypeptidase A inhibitor. Mol Biol Rep.

[2] Kouri JB, Aguilera JM, Reyes J, Lozoya KA, Gonzalez S (2000). Apoptotic chondrocytes from osteoarthrotic human articular cartilage and abnormal calcification of subchondral bone. J Rheumatol.

[3] Silver FH, Bradica G, Tria A (2001). Relationship among biomechanical, biochemical, and cellular changes associated with osteoarthritis. Crit Rev Biomed Eng.

[4] Aigner T, McKenna L (2002). Molecular pathology and pathobiology of osteoarthritic cartilage. Cell Mol Life Sci.

[5] Kouri JB, Lavalle C (2006). Do chondrocytes undergo “activation” and “transdifferentiation” during the pathogenesis of osteoarthritis? A review of the ultrastructural and immunohistochemical evidence. Histol Histopathol.

[6] Ruiz-Romero C, Lopez-Armada MJ, Blanco FJ (2006). Mitochondrial proteomic characterization of human normal articular chondrocytes. Osteoarthritis Cartilage.

[7] Lozoya KA, Flores JB (2000). A novel rat osteoarthrosis model to assess apoptosis and matrix degradation. Pathol Res Pract.

[8] Ruiz-Romero C, Lopez-Armada MJ, Blanco FJ (2005). Proteomic characterization of human normal articular chondrocytes: a novel tool for the study of osteoarthritis and other rheumatic diseases. Proteomics.

[9] Martel-Pelletier J, McCollum R, Fujimoto N, Obata K, Cloutier JM, Pelletier JP (1994). Excess of metalloproteases over tissue inhibitor of metalloprotease may contribute to cartilage degradation in osteoarthritis and rheumatoid arthritis. Lab Invest.

[10] Hardingham TE, Fosang AJ (1992). Proteoglycans: many forms and many functions. FASEB J.

[11] Goldring MB (2000). The role of the chondrocyte in osteoarthritis. Arthritis Rheum.

[12] Hermansson M, Sawaji Y, Bolton M, Alexander S, Wallace A, Begum S, Wait R, Saklatvala J (2004). Proteomic analysis of articular cartilage shows increased type II collagen synthesis in osteoarthritis and expression of inhibin betaA (activin A), a regulatory molecule for chondrocytes. J Biol Chem.

[13] Vincourt JB, Lionneton F, Kratassiouk G, Guillemin F, Netter P, Mainard D, Magdalou J (2006). Establishment of a reliable method for direct proteome characterization of human articular cartilage. Mol Cell Proteomics.

[14] Belluoccio D, Wilson R, Thornton DJ, Wallis TP, Gorman JJ, Bateman JF (2006). Proteomic analysis of mouse growth plate cartilage. Proteomics.

[15] Wilson R, Bateman JF (2008). A robust method for proteomic characterization of mouse cartilage using solubility-based sequential fractionation and two-dimensional gel electrophoresis. Matrix Biol.

[16] Guo D, Tan W, Wang F, Lv Z, Hu J, Lv T, Chen Q, Gu X, Wan B, Zhang Z (2008). Proteomic analysis of human articular cartilage: identification of differentially expressed proteins in knee osteoarthritis. Joint Bone Spine.

[17] Wu J, Liu W, Bemis A, Wang E, Qiu Y, Morris EA, Flannery CR, Yang Z (2007). Comparative proteomic characterization of articular cartilage tissue from normal donors and patients with osteoarthritis. Arthritis Rheum.

[18] Perez E, Gallegos JL, Cortes L, Calderon KG, Luna JC, Cazares FE, Velasquillo MC, Kouri JB, Hernandez FC (2010). Identification of latexin by a proteomic analysis in rat normal articular cartilage. Proteome Sci.

[19] Almonte-Becerril M, Navarro-Garcia F, Gonzalez-Robles A, Vega-Lopez MA, Lavalle C, Kouri JB (2010). Cell death of chondrocytes is a combination between apoptosis and autophagy during the pathogenesis of Osteoarthritis within an experimental model. Apoptosis.

[20] Bendele AM (2001). Animal models of osteoarthritis. J Musculoskelet Neuronal Interact.

[21] Ostergaard K, Petersen J, Andersen CB, Bendtzen K, Salter DM (1997). Histologic/histochemical grading system for osteoarthritic articular cartilage: reproducibility and validity. Arthritis Rheum.

[22] Martinez-Calleja A, Velasquillo C, Vega-Lopez M, Arellano-Jimenez MJ, Tsutsumi-Fujiyoshi VK, Mondragon-Flores R, Kouri-Flores JB (2014). Osteopontin expression and localization of Ca++ deposits in early stages of osteoarthritis in a rat model. Histol Histopathol.

[23] Mankin HJ, Dorfman H, Lippiello L, Zarins A (1971). Biochemical and metabolic abnormalities in articular cartilage from osteo-arthritic human hips. II. Correlation of morphology with biochemical and metabolic data. J Bone Joint Surg Am.

[24] Mankin HJ (1971). Biochemical and metabolic aspects of osteoarthritis. Orthop Clin North Am.

[25] Kouri-Flores JB, Abbud-Lozoya KA, Roja-Morales L (2002). Kinetics of the ultrastructural changes in apoptotic chondrocytes from an osteoarthrosis rat model: a window of comparison to the cellular mechanism of apoptosis in human chondrocytes. Ultrastruct Pathol.

[26] Somech R, Lev A, Simon AJ, Hanna S, Etzioni A (2012). T- and B-cell defects in a novel purine nucleoside phosphorylase mutation. J Allergy Clin Immunol.

[27] Pagaev RM, Kakuev DL, Pozdeev VI, Kutuzov MA, Rakitina TV, Lipkin VM (2012). The light chain of the dynein complex DYNLRB1 interacts with NDP-kinase a from bovine retina. Dokl Biochem Biophys.

[28] Toro A, Grunebaum E (2006). TAT-mediated intracellular delivery of purine nucleoside phosphorylase corrects its deficiency in mice. J Clin Invest.

[29] Tokarska-Schlattner M, Boissan M, Munier A, Borot C, Mailleau C, Speer O, Schlattner U, Lacombe ML (2008). The nucleoside diphosphate kinase D (NM23-H4) binds the inner mitochondrial membrane with high affinity to cardiolipin and couples nucleotide transfer with respiration. J Biol Chem.

[30] Eyre DR, Wu JJ, Woods PE (1991). The cartilage collagens: structural and metabolic studies. J Rheumatol Suppl.

[31] Eyre DR, Weis MA, Wu JJ (2006). Articular cartilage collagen: an irreplaceable framework?. Eur Cell Mater.

[32] Horikawa O, Nakajima H, Kikuchi T, Ichimura S, Yamada H, Fujikawa K, Toyama Y (2004). Distribution of type VI collagen in chondrocyte microenvironment: study of chondrons isolated from human normal and degenerative articular cartilage and cultured chondrocytes. J Orthop Sci.

[33] Yeung K, Janosch P, McFerran B, Rose DW, Mischak H, Sedivy JM, Kolch W (2000). Mechanism of suppression of the Raf/MEK/extracellular signal-regulated kinase pathway by the raf kinase inhibitor protein. Mol Cell Biol.

[34] Chin D, Means AR (2000). Calmodulin: a prototypical calcium sensor. Trends Cell Biol.

[35] Shimazaki A, Wright MO, Elliot K, Salter DM, Millward-Sadler SJ (2006). Calcium/calmodulin-dependent protein kinase II in human articular chondrocytes. Biorheology.

[36] Sekito A, Koide-Yoshida S, Niki T, Taira T, Iguchi-Ariga SM, Ariga H (2006). DJ-1 interacts with HIPK1 and affects H2O2-induced cell death. Free Radic Res.

[37] Takahashi K, Taira T, Niki T, Seino C, Iguchi-Ariga SM, Ariga H (2001). DJ-1 positively regulates the androgen receptor by impairing the binding of PIASx alpha to the receptor. J Biol Chem.

[38] Shinbo Y, Taira T, Niki T, Iguchi-Ariga SM, Ariga H (2005). DJ-1 restores p53 transcription activity inhibited by Topors/p53BP3. Int J Oncol.

[39] Yoda M, Sakai T, Mitsuyama H, Hiraiwa H, Ishiguro N (2011). Geranylgeranylacetone suppresses hydrogen peroxide-induced apoptosis of osteoarthritic chondrocytes. J Orthop Sci.

[40] Dycus DL, Au AY, Grzanna MW, Wardlaw JL, Frondoza CG (2013). Modulation of inflammation and oxidative stress in canine chondrocytes. Am J Vet Res.

[41] Soares DC, Abbott CM (2013). Highly homologous eEF1A1 and eEF1A2 exhibit differential post-translational modification with significant enrichment around localised sites of sequence variation. Biol Direct.

[42] Kadouchi I, Sakamoto K, Tangjiao L, Murakami T, Kobayashi E, Hoshino Y, Yamaguchi A (2009). Latexin is involved in bone morphogenetic protein-2-induced chondrocyte differentiation. Biochem Biophys Res Commun.

[43] Arimatsu Y, Nihonmatsu I, Hatanaka Y (2009). Localization of latexin-immunoreactive neurons in the adult cat cerebral cortex and claustrum/endopiriform formation. Neuroscience.

[44] Liang Y, Van Zant G (2008). Aging stem cells, latexin, and longevity. Exp Cell Res.

[45] Pallares I, Bonet R, Garcia-Castellanos R, Ventura S, Aviles FX, Vendrell J, Gomis-Ruth FX (2005). Structure of human carboxypeptidase A4 with its endogenous protein inhibitor, latexin. Proc Natl Acad Sci U S A.

[46] Silvestro L, Viano I, Naggi A, Torri G, Da Col R, Baiocchi C (1992). High-performance liquid chromatographic-mass spectrometric analysis of oligosaccharides from enzymatic digestion of glycosaminoglycans. Application to human samples. J Chromatogr.

[47] Hanna SL, Sherman NE, Kinter MT, Goldberg JB (2000). Comparison of proteins expressed by Pseudomonas aeruginosa strains representing initial and chronic isolates from a cystic fibrosis patient: an analysis by 2-D gel electrophoresis and capillary column liquid chromatography-tandem mass spectrometry. Microbiology.

