# Supplementary figures and images for: Proteomic analysis of rat cartilage: the identification of differentially expressed proteins in the early stages of osteoarthritis
Source: Proteome Sci. 2014 Nov 18;12:55. doi: 10.1186/s12953-014-0055-0 (PMC4246440; doi:10.1186/s12953-014-0055-0)

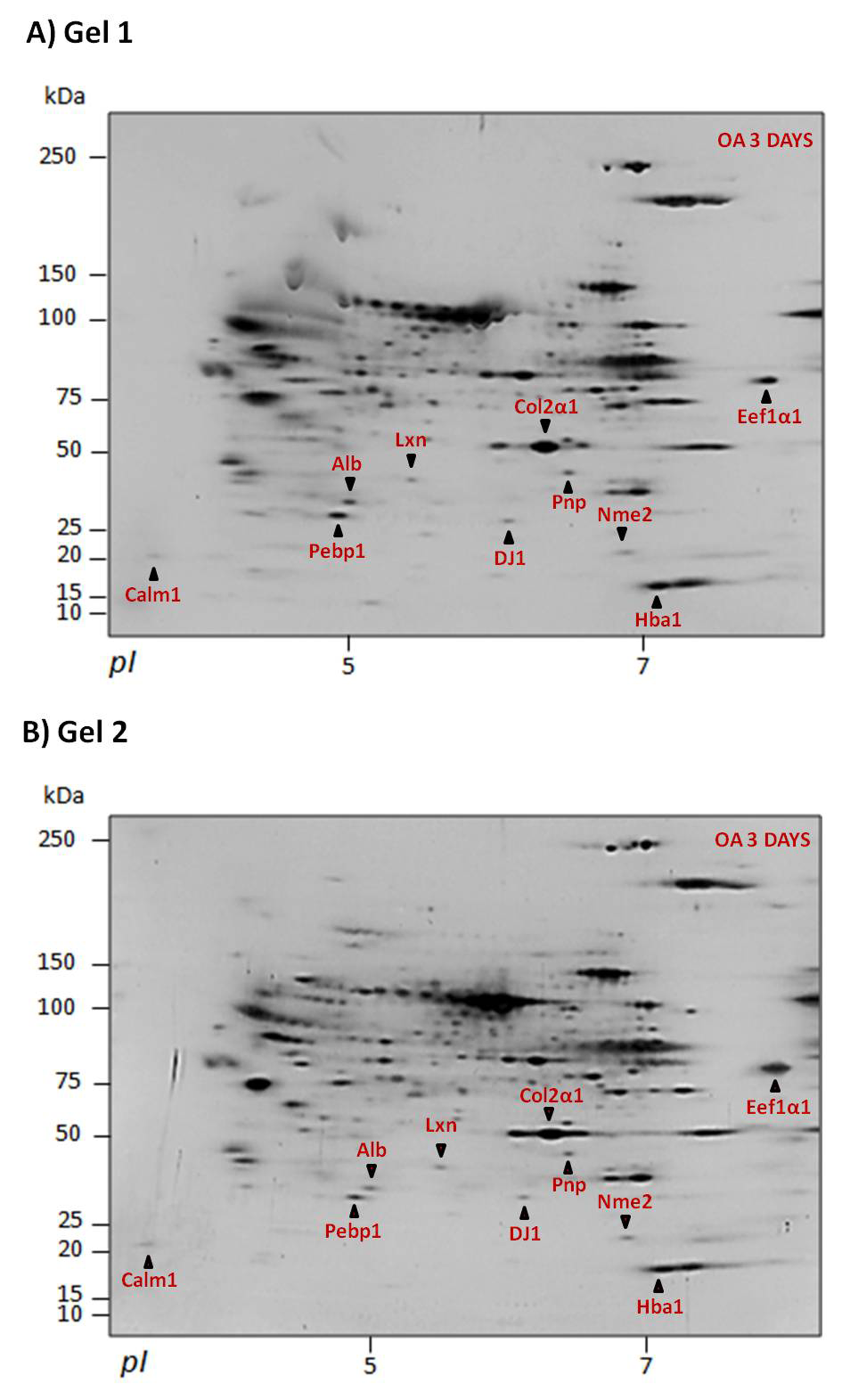

Supplement: Additional file 1: Figure S1. — Representative 2-DE proteomic maps of identified proteins in OA cartilage during early stages (3, 5, and 10 days). (A-B) Samples of OA cartilage at 3 days (duplicate experiment). Proteins were resolved on IPG strips pH 3–10 NL (5-20% SDS-PAGE gradient gels, gels were silver-stained). The spots were marked with arrowheads according to the database [UniProt Knowledge base (UniProtKB)/Swiss-Prot] for Rattus norvegicus species. [file 12953_2014_55_MOESM1_ESM.tiff]
